# Antiglomerular Basement Membrane Disease in a Pediatric Patient: A Case Report and Review of the Literature

**DOI:** 10.1155/2017/1256142

**Published:** 2017-05-09

**Authors:** Vimal Master Sankar Raj, Diana Warnecke, Julia Roberts, Sarah Elhadi

**Affiliations:** ^1^Department of Pediatric Nephrology, University of Illinois College of Medicine at Peoria, Peoria, IL, USA; ^2^Department of Pediatrics, University of Illinois College of Medicine at Peoria, Peoria, IL, USA

## Abstract

Goodpasture's syndrome (GPS) remains a very rare disease entity in the pediatric population characterized by the presence of pulmonary hemorrhage and rapidly evolving glomerulonephritis. We hereby describe the case of a 2-year-old girl who presented with renal failure and was diagnosed with GPS. A brief review of the literature in regard to data on demographics, pathogenesis, clinical features, diagnosis, treatment, and prognosis for renal recovery is also provided.

## 1. Introduction

Goodpasture's syndrome (GPS) is a rare and life threatening autoimmune condition with autoantibodies directed against the glomerular basement membrane (GBM) antigen. The term GPS refers to the triad of pulmonary hemorrhage, glomerulonephritis, and anti-GBM antibodies while Goodpasture's disease (GD) is the preferred terminology in the absence of pulmonary hemorrhage [1, 2]. The term antiglomerular basement membrane antibody disease (aGD) describes a patient with serum antibodies against the basement membrane and includes both Goodpasture's syndrome and disease.

## 2. Case Report

A 2-year, 11-month-old Hispanic female presented to her primary care physician's office with swelling of the hands and face following one week of fever, sore throat, and malaise. A screening urine analysis (U/A) revealed 3+ protein and blood with numerous red blood cells per high power field. Further work-up also demonstrated anemia (Hb of 8.5 g/dl) and several electrolyte imbalances with azotemia (BUN 116 mg/dl and Cr 7.3 g/dl) prompting immediate transfer to our Children's Hospital for further evaluation and management.

On admission, examination revealed a pale child with bilateral mild pedal edema and blood pressure of 125/71 mm Hg. She was afebrile, mildly tachycardic, and saturating at 100% in room air and parents denied any h/o joint pain/swelling or skin rash. Her urine output was noted to be darker and less frequent over the past few days.

**Figure 1:**
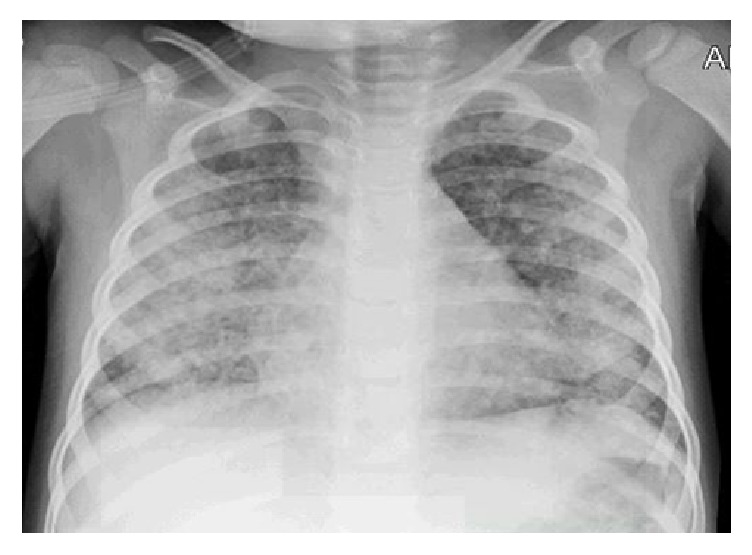
Chest X-ray on initial presentation.

Past medical history was significant for an admission about 7 months back with respiratory distress and presumed pneumonia. Labs at that time were significant for severe anemia (Hb 6.9 g/dl) and iron deficiency. Initial chest X-ray showed bilateral diffuse peribronchial cuffing and nodular opacities with concerns for severe bronchiolitis/bronchopneumonia (Figure 1). Patient was started on empiric antibiotic coverage but respiratory distress worsened to the point of requiring ventilator support. A work-up at that time showed elevated erythrocyte sedimentation rate (113 mm/hr) and negative serology for antinuclear antibody. A respiratory viral pathogen array came back positive for rhino virus. Patient's clinical condition continued to deteriorate and patient was placed on extracorporeal membrane oxygenation (ECMO). During this time patient was also started on high dose methylprednisolone with presumptive exaggerated inflammatory response to her viral pneumonia in a bid to reduce inflammation. Patient had a dramatic response to steroids and was off ECMO in 2 days and off ventilator support within a week time. A U/A done during this past hospital stay showed 1+ blood and no protein and a chemistry panel showed normal renal function with a serum creatinine of 0.41 mg/dl.

Work-up during this current admission confirmed anemia (Hb 7.8 g/dl) with a slightly elevated white blood cell count (10,400/mm3) and platelet count (234,000/mm3). Serum chemistry panel was abnormal for hyperkalemia (6 mmol/L), metabolic acidosis (Hco3 of 8 mmol/L), hypocalcemia (5.6 mg/dl), hyperphosphatemia (8.9 mg/dl), and renal failure (BUN 120 mg/dl and Cr 7.01 mg/dl). Further work-up involved evaluation as to identify the cause of glomerulonephritis and showed normal complement levels, normal coagulation profile, negative serology for viral etiology, lupus, and ANCA titers. Parathyroid hormone levels were elevated indicating a state of chronic kidney damage. ESR was elevated at 24 mm/hr but lesser than the prior admission value of 113 mm/hr and a CRP was not checked during the current admission. Urine protein to creatinine ratio was in the nephrotic range and antiglomerular basement membrane (GBM) titers were sent. Urine output recorded was between 1.5 and 2 ml/kg/hr during the initial few days but progressively got oliguric (0.3 to 0.5 ml/kg/hr) from the first week onwards. Renal ultrasound showed normal sized kidneys (right and left kidney around 7.2 cm) with increased cortical echogenicity bilaterally. A comparison of lab values during prior hospital stay and current admission is provided in Table 1.

**Figure 2:**
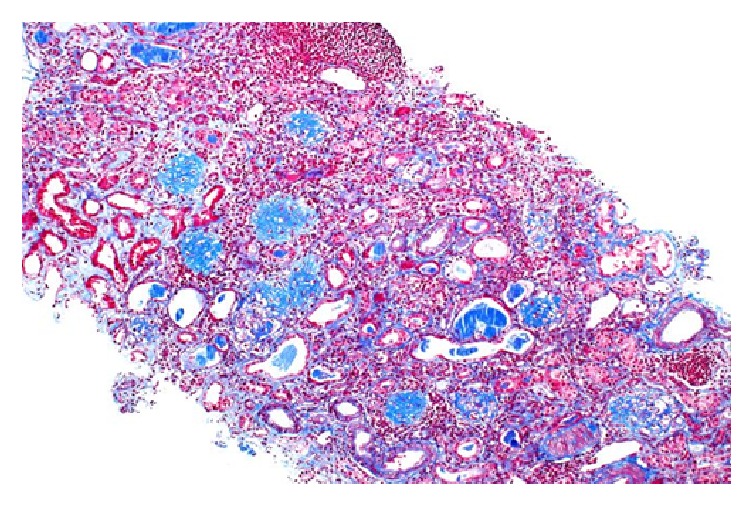
Trichrome stain showing global glomerulosclerosis.

**Figure 3:**
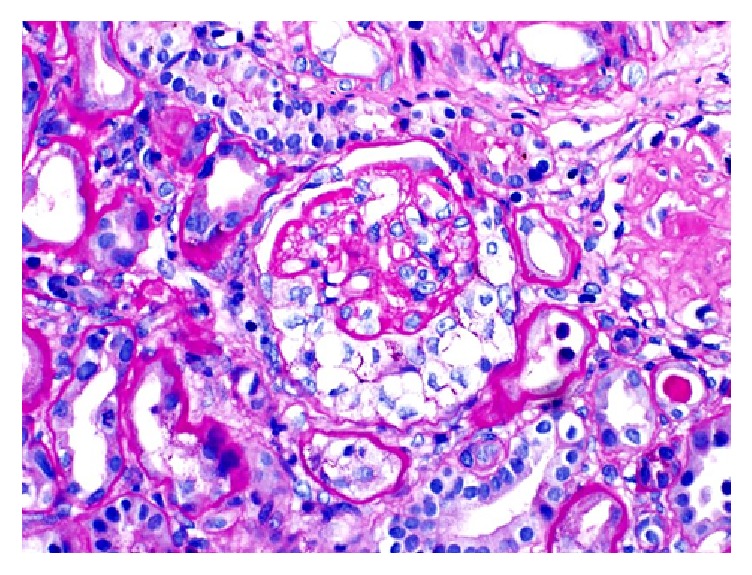
Glomerulus showing intraglomerular sclerosis.

**Figure 4:**
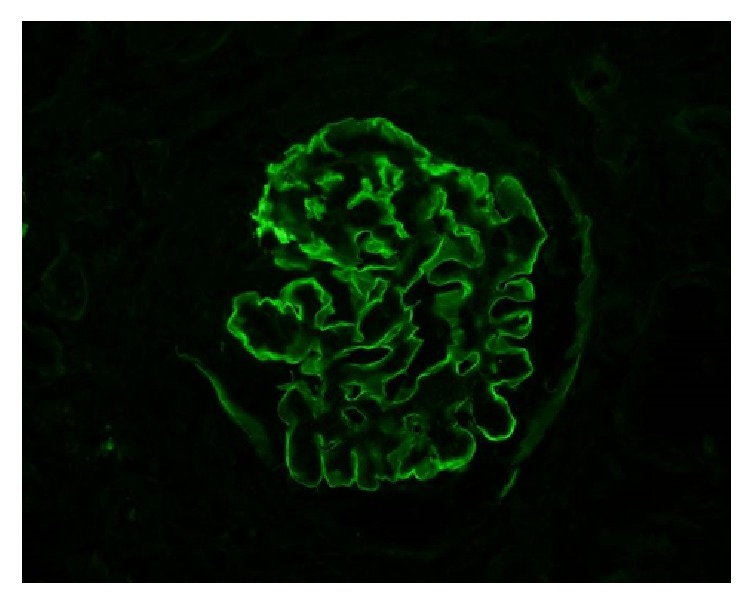
Immunofluorescence showing linear IgG deposits.

Patient underwent emergent hemodialysis to correct electrolyte imbalances. We proceeded with a renal biopsy to ascertain a tissue diagnosis for the glomerulonephritis. 12 glomeruli were available for light microscopic examination. 9/12 glomeruli showed global sclerosis (Figure 2). Remaining glomeruli showed cellular to fibrocellular crescents (Figure 3). The interstitium is involved by a dense inflammatory infiltrate composed of lymphocytes, plasma cells, and scattered eosinophils. No definite granulomas were identified. Immunofluorescence showed intense linear glomerular capillary staining with IgG, Kappa, and lambda chains (Figure 4). The renal biopsy findings were consistent with anti-GBM mediated crescentic glomerulonephritis.

Patient was started on high dose methylprednisolone and plasmapheresis once the biopsy results were consistent with anti-GBM disease. Anti-GBM titers (IgG antibody) also came back elevated at 1.1 units (Normal < 1) confirming the diagnosis. The subtle elevation in anti-GBM titers could be secondary to the possibility of a serological remission though chronic damage to the kidneys has already happened as documented by the amount of fibrosis on renal biopsy. Unfortunately anti-GBM titers were not checked during the initial pneumonia like presentation 7 months back which likely represented the initial acute episode. With every other day plasmapheresis, anti-GBM titers started trending down but renal function did not recover. With the extent of global sclerosis noted in renal biopsy and with the very high PTH levels, chances for renal recovery remained slim. Rituximab was used as an alternate immunosuppressive agent instead of cyclophosphamide taking into consideration the amount of chronic damage noted on renal biopsy in an attempt to reduce infectious risk. Patient received a total of 5 sessions of plasmapheresis with no improvement in renal function and was transitioned to peritoneal dialysis. During the inpatient stay she suffered a hypertensive crisis with seizures and the control of blood pressure required multiple antihypertensive agents. Anti-GBM titers were periodically monitored by lab work on a monthly basis and remained negative on maintenance immunosuppression with mycophenolate and low dose prednisone. Patient received a diseased donor kidney transplant, 2 months back, and is currently doing well with normal renal function.

## 3. Discussion

### 3.1. Epidemiology

GPS is a rare condition occurring in approximately 0.5 to 1 per million per year in adults and even more rare in children [3]. According to the United States Renal Data Registry, incidence of pediatric end stage renal disease (ESRD) due to this rare entity is only 11-12 per year, accounting for 0.5% of pediatric ESRD in 2009–2013 [4]. It typically has a bimodal distribution with the first peak predominantly affecting males in their teens and twenties. The second peak which happens in older population (>60 years of age) affects male and female equally. GPS is rare in children, with only about 30 cases being reported in the pediatric literature, with the youngest reported child being 11 months of age. The previously reported cases of pediatric Goodpasture's syndrome over the past 25 years are detailed in Table 2 [5–13].

### 3.2. Pathogenesis

The type IV collagen which provides the backbone for GBM formation is the target for autoantibody formation and damage in GPS. The type IV collagen has six genetically discrete chains (*α*1 to *α*6) which are arranged into triple helical protomers (*α*1*α*1*α*2, *α*3*α*4*α*5, and *α*5*α*5*α*6) of varying composition. The protomer has a 7S domain at the N-terminal, a collagenous part in the middle, and a noncollagenous (NC1) domain at the C-terminal [14]. The final collagen IV network in the GBM is a polymerized mesh such that the 7S domain forms a tetramer and the NC1 domain forms a hexamer providing the tensile strength to the basement membrane. *α*1*α*1*α*2-*α*1*α*1*α*2 is the predominant collagen prototype in embryonic GBM and a developmental switch happens to the final adult form of *α*3*α*4*α*5-*α*3*α*4*α*5 anywhere between 3 months and 3 years of age [15].

The specific target for autoantibody formation in GPS is the NC1 domain of the *α*3 subunit in the C-terminal. The NC1 domain also acts as the main promoter for collagen polymerization. The common presence of *α*3 collagen in the basement membrane of both kidneys and lungs explains the predominant organ involvement in this condition. A triggering event (upper respiratory infection, smoking, hydrocarbon exposure, and influenza) in a genetically susceptible individual causes exposure of the *α*3 NC1 domain and subsequent antibody formation [16, 17]. Strong HLA association with presence of HLA-DR15 and DR 4 allele in about 80% of affected individuals confirms a genetic predisposition as is the case in the majority of autoimmune diseases [18]. The absence of *α*3 subunit in younger children (before the developmental switch) could be attributed to the lesser incidence of aGD in younger children.

### 3.3. Clinical Features

Initial presentation of GPS can be nonspecific and often consists of symptoms such as malaise, weight loss, fever, and arthralgia [19]. Kidney disease may occur independently or with pulmonary disease. Renal manifestations vary widely and can range from hematuria and proteinuria to rapidly progressing renal failure with oliguria, fluid overload, and severe hypertension. Pulmonary symptoms may precede renal symptoms by weeks to months with hemoptysis being the most common pulmonary manifestation. Pulmonary bleeding can be occult leading to anemia and iron deficiency but the usual presentation is with profound pulmonary hemorrhage causing respiratory failure and death in a matter of hours. Other organ system involvement is very rare though cerebral vasculitis with confusion, aphasia, and seizures has been reported in the literature [20].

### 3.4. Pathology

The diagnosis of antiglomerular basement membrane disease is reliant on detection of anti-GBM antibodies either in circulation or in the tissue by means of renal or pulmonary biopsies. Serological testing for anti-GBM antibody titers (IgG1 subclass) usually employs ELISA methodology. The sensitivity of available commercial kits can vary from 63% to 100% underlying the possibility of missed diagnosis if solely reliant on serological testing [21, 22]. Renal biopsy can help confirm the diagnosis of GPS and also provides important clues on the amount of chronicity/activity helping to guide treatment. Light microscopy usually shows crescentic glomerulonephritis but the characteristic linear IgG deposition along the capillary wall is noted in immunofluorescence microscopy clinching the diagnosis. Lung biopsy also shows the linear IgG deposits but this finding is not as constant as in kidney [23].

### 3.5. Treatment

Early diagnosis is important in terms of ability to recover renal function. Treatment of choice initially is plasmapheresis to remove circulating antibodies. The preferred immunosuppressive therapy includes corticosteroids and cyclophosphamide to reduce antibody production. Alternate immunosuppressive therapy including rituximab has been tried in resistant cases [24]. Anti-GBM antibodies are monitored weekly until two negatives are achieved, at which time levels are monitored monthly for up to 6 months. Low dose prednisone, azathioprine, or mycophenolate may be used for maintenance immunosuppression once remission is established with cessation of antibody production. If the antibody titer levels remain positive, the immunosuppression therapy should be continued [25].

### 3.6. Prognosis

Unfortunately, many patients die secondary to pulmonary hemorrhage or renal failure before plasmapheresis and immunosuppression can be initiated. Currently the mortality rate is 20% in adults and 30% in children. Prognosis for renal recovery is worse in the presence of oliguria, presenting creatinine >6.8 or renal biopsy showing >50% crescent formation within glomeruli at time of diagnosis [26]. Evidences of chronic damage as documented by moderate or severe interstitial fibrosis and global glomerulosclerosis always carry a worse prognosis. Renal outcome is dependent on timing of diagnosis with improved outcomes if treatment is initiated within 4 weeks of renal involvement [27]. Despite the potential seriousness of lung hemorrhage with increased fatality, no residual pulmonary deficit or fibrosis is noted once patient recovers from the acute presentation. Though rare, recurrence of disease may occur years after initial presentation.

GPS as a cause of pulmonary renal syndrome in childhood remains extremely rare. A review of pediatric cases in the literature (Table 2) shows a female preponderance in children in comparison to majority male involvement in adults. Anti-GBM titers were positive in almost all of the reported cases. Majority of cases also showed dominant renal involvement with gross hematuria and oligoanuria being the most common presentation. Treatment strategies involved using a combination of steroids, plasma exchange, and cyclophosphamide in most of the patients. Renal recovery was noted only in 3/11 patients among whom one had presentation with nephrotic range proteinuria but with normal renal function [8]. The prognosis for renal recovery seems to be better in the absence of interstitial fibrosis and early treatment initiation as is noted in the adult literature.

## 4. Conclusion

In conclusion, GPS is a rare autoimmune condition presenting with significant mortality and morbidity in children. We report a case of 2-year, 11-month-old child who presented with this condition and the difficulties involved in coming to an accurate diagnosis. In hindsight, her initial presentation with pneumonia was likely an occult pulmonary hemorrhage as documented by the severe anemia and iron deficiency. Her response to steroids during the initial admission likely constituted a partial treatment. GPS though rare should be considered in the differential diagnosis of clinical presentation with lung and kidney involvement and early diagnosis and intervention are essential for a favorable outcome.

## Figures and Tables

**Table 1:** Comparison of lab values between prior and current admission.

Labs	10/2015	5/2016
Sodium (mmol/L)	141	137
Potassium (mmol/L)	4.2	6.0
Chloride (mmol/L)	110	113
Carbon dioxide (mmol/L)	21	8
Glucose (mg/dL)	109	97
BUN (mg/dL)	5	120
Creatinine (mg/dL)	0.41	7.01
Albumin (g/dL)	2.9	2.5
Calcium (mg/dL)	9.3	5.6
Phosphorus (mg/dL)		8.9
White blood cell (×10^3^/mcL)	15.84	10.4
Hemoglobin (g/dL)	6.9	7.8
Hematocrit (%)	22.6	23.7
Platelets (×10^3^/mcL)	616	234
Ferritin (ng/mL)	269	267
Iron (mcg/dL)	6	55
Transferrin (mcg/dL)	130	100
TIBC (mcg/dL)	Not done	125
% saturation	Not done	44
Parathyroid hormone (pg/mL)	Not done	1031
C3 (mg/dL)	166	114
C4 (mg/dL)	30	48
ESR (mm/hr)	113	12.5
CRP (mg/dl)	12.5	Not done

**Table 2:** Prior reported cases of pediatric Goodpasture's syndrome.

Age in years	Sex	Anti-GBM titers	Initial clinical presentation	Renal biopsy	Renal outcome	Pulmonary outcome	Treatment	Final outcome	Reference
4	F	Positive	Pallor, fatigue oliguria, proteinuria, and microscopic hematuria with dominant renal involvement	End stage glomerulonephritis with crescent formation; linear deposition of IgG along basement membrane	No improvement	Stable	Prednisone, azathioprine, and cyclophosphamide	Died	[5]
10	F	Positive	Gross hematuria, oliguria, and uremia with dominant renal involvement Preceding infection with strep throat	Endocapillary and extracapillary proliferative GN with 80% crescents Immunofluorescence could not be done	Dialysis dependent with no improvement	Stable	Prednisolone, azathioprine, and plasmapheresis	Remained dialysis dependent	[5]
7	F	Positive	Diarrhea, vomiting, oliguria, and pallor with dominant renal involvement	Crescentic nephritis with linear IgG deposition	Initial improvement in urine output and GFR with subsequent decline and dialysis dependence	Stable	Plasmapheresis, prednisolone, and cyclophosphamide	Dialysis dependent	[5]
6	M	Positive	Dominant renal involvement	Diagnostic with crescentic nephritis	Improved	Stable	Steroid, plasmapheresis, and immunosuppression	Regained renal function	[6]
10	M	Positive	Cough, right lower lobe infiltrate, vomiting, and oliguria with dominant pulmonary involvement and pulmonary hemorrhage	Crescentic nephritis with extensive necrosis	Deterioration in renal function with dialysis dependence	Improved	Steroid, plasmapheresis, and immunosuppression	Dialysis dependent	[7]
2.5	F	Positive	Fever, anorexia with *E. coli* UTI as initial presentation with worsening renal function and oliguria	Extensive crescentic necrotizing nephritis with linear IgG deposits	No improvement	Stable	Steroid, plasmapheresis, and immunosuppression	Dialysis dependent	[8]
11 months	F	Positive	Dominant renal involvement	Diagnostic with crescentic nephritis	No improvement	Stable	Steroid, plasmapheresis, and immunosuppression	Renal transplant	[9]
5.6	F	Positive	Fever, malaise, and gross hematuria with rapid decline in renal function	Diffuse cellular crescentic nephritis with linear IgG deposits	Recovery of renal function	Stable	Plasma exchange, solumedrol, and Cytoxan	CKD with stable renal function	[10]
9	M	Positive	Malaise, anorexia, and oligoanuria with pulmonary hemorrhage	Not done	Not improved	Pulmonary status improved	Plasma exchange, solumedrol, and Cytoxan	Dialysis dependent	[11]
8	F	Positive	Asymptomatic with persistent nephrotic range proteinuria and microhematuria	No crescents but with linear deposits of IgG	Improvement in proteinuria with stable renal function	Stable	Plasma exchange, prednisone, and oral Cytoxan	Asymptomatic	[12]
19 months	M	Positive	Gross hematuria, proteinuria with rapid decline in renal function	Crescentic GN with weak global linear staining of IgG	Improvement in proteinuria and renal function	Stable	Plasma exchange, solumedrol, and Cytoxan	Asymptomatic	[13]
